# Complete Genome Sequence of Stenotrophomonas maltophilia Siphophage Summit

**DOI:** 10.1128/mra.00089-22

**Published:** 2022-03-07

**Authors:** Alexandra Bishop, Xing-Han Zhang, James Clark, Tram Le, Ben Burrowes, Mei Liu

**Affiliations:** a Department of Biochemistry and Biophysics, Texas A&M University, College Station, Texas, USA; b Center for Phage Technology, Texas A&M University, College Station, Texas, USA; Queens College CUNY

## Abstract

Stenotrophomonas maltophilia is an opportunistic pathogen exhibiting resistance to multiple antimicrobials. This study reports the complete genome of an S. maltophilia siphophage, Summit. Its genome of 95,728 bp has 148 protein-coding genes and 5 tRNAs, including 1 predicted suppressor tRNA. Possible target genes for the suppressor tRNA are not identified.

## ANNOUNCEMENT

Stenotrophomonas maltophilia, a Gram-negative bacterium, is both a human opportunistic pathogen and potential biocontrol agent for use in bioremediation (1, 2). S. maltophilia exhibits intrinsic resistance to various classes of antimicrobials, causing the need for new drugs, such as bacteriophage therapy, to counter S. maltophilia infections (2, 3). This report summarizes the complete genome annotation of a novel S. maltophilia phage siphophage, Summit.

Bacteriophage Summit was isolated from a weaning foal swab sample provided by the Texas A&M Veterinary Clinic, College Station, TX. The phage was isolated and cultured by the soft agar overlay method (4) with S. maltophilia (ATCC 17807) as a propagation host grown aerobically at 30°C in tryptone nutrient broth. Following purification, phage DNA was isolated as previously described (5), prepared as 300-bp inserts using a Swift 2S Turbo kit, and sequenced on an Illumina MiSeq with pair-end 150-bp reads using a V2 300-cycle chemistry. Using FastQC (www.bioinformatics.babraham.ac.uk/projects/fastqc) and FASTX-Toolkit v0.0.14 (http://hannonlab.cshl.edu/fastx_toolkit/) resulted in 129,787 trimmed reads. Summit’s genome was then assembled by SPAdes v3.5.0 into a single contig with 88-fold coverage (6). Forward (5′-TGGAAGAGAAGGCCACGAAC-3′) and reverse (5′-CCGAGTCGAGGTAGAACGTG-3′) closure primers were used to close the genome by PCR and Sanger sequencing. The genome was annotated using the CPT Galaxy-Apollo phage annotation platform (7–9). Structural annotation was completed using Glimmer v3 (10) and MetaGeneAnnotator v1.0 (11). tRNAs were identified with ARAGORN v2.36 (12) and tRNAScan-SE v2.0 (13). Functional gene annotation was completed using InterProScan v5.48 (14), BLAST v2.9.0 (15), TMHMM v2.0 (16), HHPred (17), LipoP v1.0 (18), and SignalP v5.0 (19). BLAST comparison was run against two databases, NCBI nr and SwissProt (20). Genome-wide DNA sequence similarities were calculated by ProgressiveMauve v2.4 (21). All analyses were done at default settings. Phage morphology was determined by staining samples with 2% (wt/vol) uranyl acetate (22) and viewing the samples by transmission electron microscopy (TEM) at the Texas A&M Microscopy and Imaging Center.

**FIG 1:**
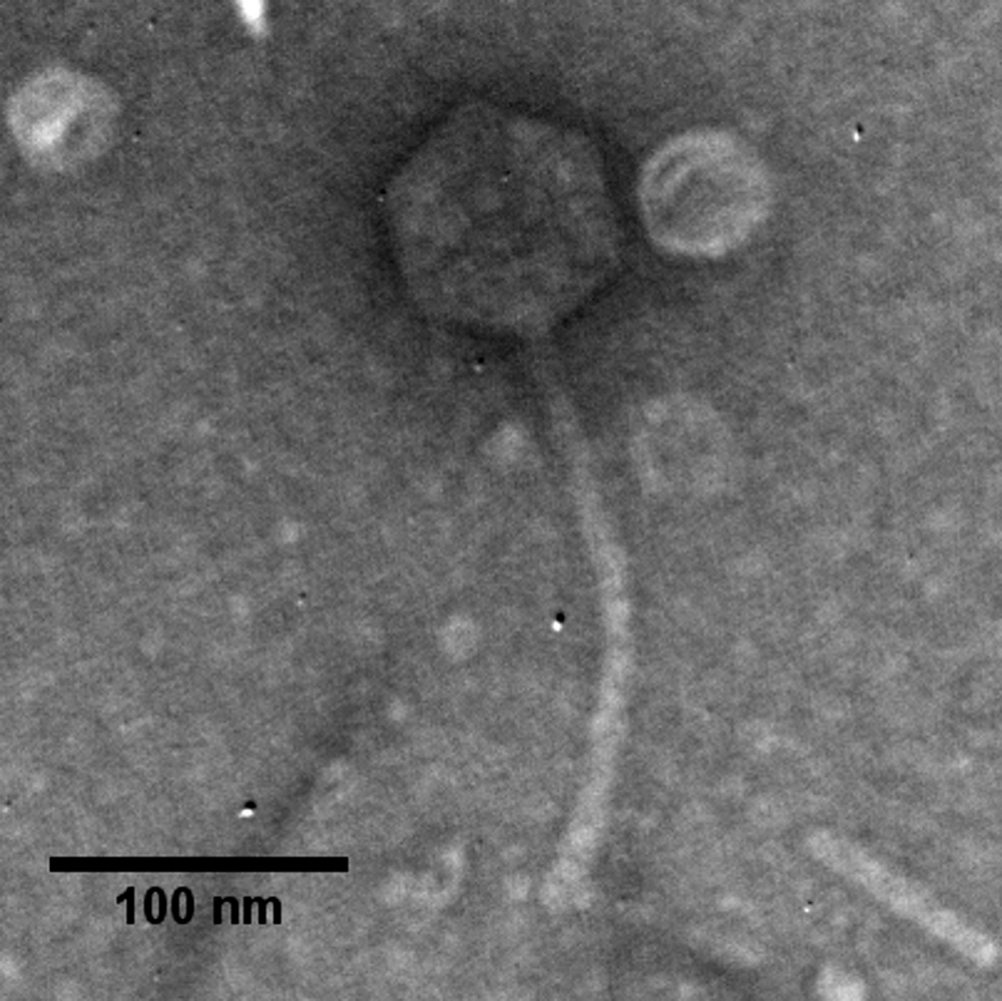
Transmission electron micrograph (TEM) of phage Summit. Phage particles were diluted with TEM buffer (20 mM NaCl, 10 mM Tris-HCl [pH 7.5], 2 mM MgSO_4_) and captured on freshly glow-discharged, Formvar carbon-coated grids. The grids were stained with 2% (wt/vol) uranyl acetate and observed on a JEOL 1200 EX TEM at 100-kV accelerating voltage at the Microscopy and Imaging Center at Texas A&M University.

Phage Summit has a siphophage morphology (Fig. 1). It has a genome of 95,728 bp with a 58.5% GC content and 93.7% coding density. Annotation of the genome revealed 148 protein-coding genes and 5 tRNAs, including 1 predicted suppressor tRNA. Analysis was done to identify possible target genes for the predicted amber stop codon suppressor tRNA. No significant genes were identified that would require the suppressor tRNA or that were lacking a Shine-Dalgarno sequence. There are 12 genes in the genome that have amber stop codons. None of those genes are followed by a predicted gene that was in frame that could be fused to its upstream neighbor by amber suppression, and no identifiable conserved domains or BLAST hits were found in downstream sequences that would be the product of such a readthrough. No tRNA synthetase protein was predicted during functional annotation. Therefore, the purpose of the predicted suppressor tRNA remains unknown. At the time of the analysis, *Stenotrophomonas* phage vB_SmaS_DLP_5 (GenBank accession number NC_042082.1) was the only known phage closely related to Summit, sharing 86.4% nucleotide similarity as determined by ProgressiveMauve and 139 proteins (BLASTp; E value < 0.001).

### Data availability.

Summit was deposited in GenBank with accession number MZ326862. The associated BioProject, SRA, and BioSample accession numbers are PRJNA222858, SRR14095250, and SAMN18509476, respectively.
